# Comparative efficacy & safety of buparlisib plus fulvestrant, fulvestrant plus dalpiciclib, and ribociclib plus letrozole for postmenopausal, hormone receptor-positive, and HER2-negative breast cancer

**DOI:** 10.1016/j.clinsp.2023.100291

**Published:** 2023-10-29

**Authors:** Qi Liu, Lingli Hou, Ying Zhao, Hongwei Yang, Zhengying Mo, Fei Yu

**Affiliations:** a Department of Human Anatomy, School of Basic Medical Sciences, Hubei University of Medicine, Shiyan, PR China; b Department of Clinical Laboratory, Taihe Hospital, Affiliated Hospital of Hubei University of Medicine, Shiyan, PR China; c Department of Oncology, Taihe Hospital, Affiliated Hospital of Hubei University of Medicine, Shiyan, PR China; d Department of Clinical Laboratory, People's Hospital of Yunxi County, Yunxi, PR China

**Keywords:** Breast cancer, Buparlisib, Dalpiciclib, Endocrine therapy, Fulvestrant, Letrozole, Ribociclib

## Abstract

**Objectives:**

This study aimed to compare progression-free survival, overall survival, clinical benefits, and adverse effects in postmenopausal women with hormone receptor-positive and HER2-negative breast cancer who received buparlisib plus fulvestrant against those of women who received dalpiciclib plus fulvestrant, considering ribociclib plus letrozole treatment as the reference standard.

**Methods:**

Women received buparlisib plus fulvestrant (BF cohort, *n* = 108), dalpiciclib plus fulvestrant (DF cohort, *n* = 132), or ribociclib plus letrozole (RL cohort, *n* = 150) until unacceptable toxicity was observed.

**Results:**

A total of 117 (89 %), 80 (74 %), and 84 (56 %) women in the BF, DF, and RL cohorts, respectively, had clinical benefits. After treatment, the clinical benefits for women and after 42 months of follow-up progression-free survival and overall survival were higher in the DF cohort than in the BF and RL cohorts (*p* < 0.05 for all). Neutropenia, vomiting, constipation, nausea, diarrhea, and anorexia were reported higher in women of the DF and BF cohorts than in women of the RL cohort. Leukopenia and increased levels of alanine aminotransferase and aspartate aminotransferase were reported to be higher in women in the RL cohort than in women in the DF and BF cohorts. Depression, anxiety, and increased levels of alanine aminotransferase and aspartate aminotransferase were reported to be higher in women in the BF cohort than in women in the DF and RL cohorts.

**Conclusions:**

Dalpiciclib plus fulvestrant is effective and comparatively safe in postmenopausal women with hormone receptor-positive and HER2-negative breast cancers. Dalpiciclib, buparlisib, fulvestrant, and ribociclib cause neutropenia, severe depression, adverse gastroenterological effects, and adverse hepatological effects, respectively.

## Highlights

• Dalpiciclib + fulvestrant is effective in hormone (+) and HER2 (−) breast cancers.
• Dalpiciclib and buparlisib cause neutropenia.
• Gastrointestinal tract-related adverse effects while treatment with fulvestrant.
• Liver function monitoring is recommended for ribociclib + letrozole treatment.
• Women should be under the supervision of a consultant while 100 mg/day of buparlisib.

## Introduction

Breast cancer is the most prevalent cancer in Chinese women.^1^ In breast cancer, the most common tumor subtype is hormone receptor-positive.^2^ Endocrine therapy-based regimens are the preferred treatment for hormone receptor-positive breast cancers.^3^ Ribociclib is an oral selective inhibitor of cyclin-dependent kinases 4 and 6.^4^ Ribociclib plus letrozole combination has high progression-free survival in premenopausal^5^ and postmenopausal^6^ women with hormone receptor-positive, HER2-negative, breast cancer, but has worse adverse effects, such as neutropenia and leukopenia. Women with hormone receptor-positive HER2-negative breast cancer in China are treated with fulvestrant plus CDK4/6 inhibitors (for example, dalpiciclib).^7^ In addition, palbociclib, ribociclib, and abemaciclib plus fulvestrant were approved by the United States Food and Drug Administration (USFDA) and the European Medicines Agency.^8^ The Chinese Society of Clinical Oncology Breast Cancer recommends CDK4/6 inhibitors with endocrine therapy for hormone receptor-positive HER2-negative breast cancer.^9^

Hormone receptor-positive breast cancer has various genomic alterations and is not homogeneous. Therefore, there are opportunities for targeted therapies.^10^ In hormone receptor-positive breast cancer, PIK3CA mutation activation causes disease progression and resistance to endocrine therapy.^11^ Therefore, targeting phosphatidylinositol 3-kinase is a potential therapeutic strategy.^10^ Buparlisib is an oral phosphatidylinositol 3-kinase inhibitor.^12^ Fulvestrant is a selective estrogen receptor degrader, and the combination of buparlisib with fulvestrant has favorable clinical outcomes with manageable adverse effects in women with metastatic estrogen receptor-positive breast cancer^10^^,^^13^; however, this combination (buparlisib plus fulvestrant) has the highest rate of discontinuation of treatment.^10^

The objectives of this retrospective study were to compare progression-free survival, overall survival, clinical benefits, and adverse effects in postmenopausal Chinese women with hormone receptor-positive and HER2-receptor-negative breast cancer who received buparlisib plus fulvestrant against those of women who received dalpiciclib plus fulvestrant, considering ribociclib plus letrozole treatment as the reference standard.

## Materials and methods

### Ethics approval and consent to participate

The protocols of the established study were designed by the authors and approved (Approval number: 14Y18 dated 15 January 2019) by the human ethics committee of the Taihe Hospital and the Chinese Society of Clinical Oncology Breast Cancer. The current study followed the law of China and the current version of the Declaration of Helsinki. As this was a retrospective study, informed consent to participate was waived by the human ethics committee of Taihe Hospital.

### Inclusion criteria

Postmenopausal women with confirmed (histologically or cytologically confirmed) hormone receptor-positive and HER2-negative breast cancers were included in the analysis.

### Exclusion criteria

Women with severe depression were excluded from the study.

### Cohorts

One hundred and eight women received 100 mg/day oral buparlisib^10^ plus intramuscular 500 mg fulvestrant (BF cohort). One hundred thirty-two women received oral 150 mg/day dalpiciclib^8^ plus intramuscular 500 mg fulvestrant (DF cohort). One hundred and fifty women received oral 600 mg/day ribociclib plus oral 2.5 mg/day letrozole^5^ (RL cohort). A total of oral 100 mg/day buparlisib,^10^ or 150 mg/day dalpiciclib,^8^ or 600 mg/day ribociclib^5^ was administered once daily for 3-weeks followed by a washout period of one week and with a total treatment period of (cycle) was 4-weeks. Fulvestrant was administered intramuscularly on day one, followed by day 15 of the first cycle. Then, after (after the first cycle) intramuscularly only on day 1 of the 4-week cycle.^8^ These treatment cycles were continued until unacceptable toxicity was achieved.

### Outcome measures

Eastern Cooperative Oncology Group (ECOG) performance status.

It is graded as, 0, fully active; 1, restricted strenuous activity; and ≥2, increasing disability.^14^

### Survival

Progression-free survival.

From the start of treatment(s) to the first documented progression of disease or death due to any reason, progression-free survival was considered.^10^

Overall survival.

From the start of treatment(s) to death due to any reason, it was considered as overall survival.^10^

### Clinical benefits

Clinical benefits were defined as the sum of complete response, partial response, and no signs of progressive response after treatment(s).^10^ The Response Evaluation Criteria in Solid Tumors (RECIST) version 1.1 criteria^15^ were used for the evaluation of complete response, partial response, and no signs of progressive response.

### Adverse effects

The Common Terminology Criteria for Adverse Events (CTCAE) v5.0^16^ were used to evaluate adverse events during the treatment and follow-up periods.

### Statistical analyses

Statistical analyses were performed using 3.01 InSat (GraphPad Software, San Diego, CA, USA). Categorical, continuous linear, and continuous nonlinear variables are depicted as frequencies with percentages in parentheses, mean ± Standard Deviation (SD), and medians with Q3–Q1 in parentheses, respectively. Fisher's exact test or chi-square test (*χ*^2^-test, for sample size > 40) was used for statistical analyses of categorical variables. The Kolmogorov–Smirnov method was used to check the linearity of continuous variables. One-way analysis of variance (ANOVA) was used for the statistical analyses of continuous linear variables. All results were considered statistically significant at *p* < 0.05.

## Results

### Study population

**Fig. 1:**
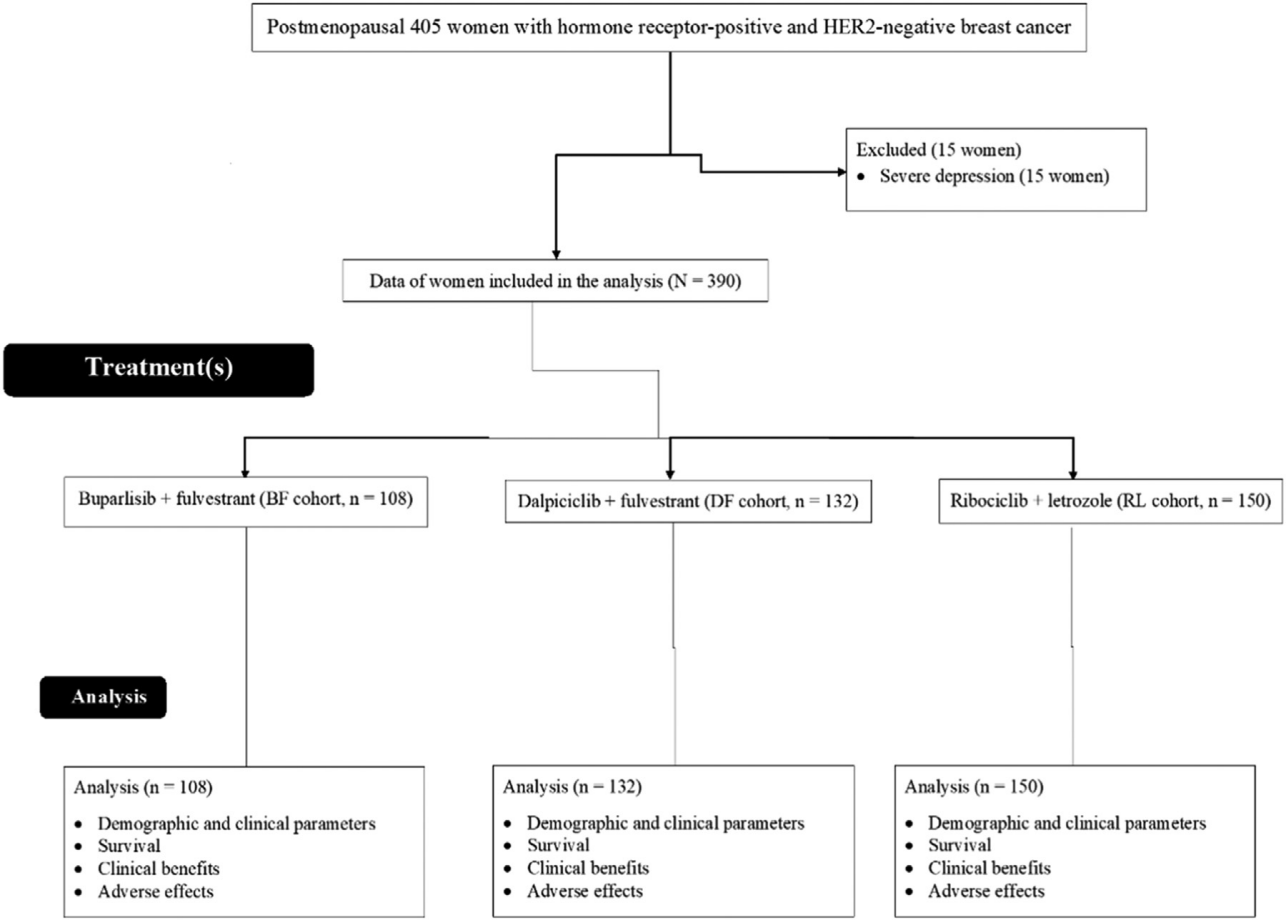
The flow chart of the retrospective analyses.

From March 1, 2017, to January 13, 2019, 405 postmenopausal women with hormone receptor-positive and HER2-negative breast cancer were treated at the Hubei University of Medicine, Shiyan, Hubei, P.R. China, the Taihe Hospital, Shiyan, Hubei, P.R. China, and the People's Hospital of Yunxi County, Yunxi, Hubei, P.R. China. Among 405 women, 15 had severe depression. Therefore, these women were excluded from this study. Survival, clinical benefits, and adverse effects in 390 postmenopausal women with hormone receptor-positive and HER2-negative breast cancer were included in the analyses. A flow chart of the retrospective analysis is shown in Fig. 1.

### Demographic and clinical parameters

All the women were approximately 50 years of age. More than 50 % of included women had an ECOG performance status of ‘0’ and more than 90 % of included women had an ECOG performance status of ‘1’ or less. Age, ethnicity, and ECOG performance status of women were comparable among the cohorts (*p* > 0.05, Table 1).

**Table 1:** Demographic and clinical parameters of women before treatment(s).

Parameters	Total	Cohorts			Comparisons between cohorts		
		BF	DF	RL			
Treatments	‒	Buparlisib+fulvestrant	Dalpiciclib+fulvestrant	Ribociclib+letrozole			
Women	390	108	132	150	*p*-value	df	Test value
Age (years)	58.35±5.36	58.08±4.57	57.92±5.63	58.91±5.62	0.2485 (one-way ANOVA)	389	1.397
Ethnicity							
Han Chinese	343 (90)	98 (91)	120 (90)	135 (90)	0.9993 (χ^2^-test)	6	0.3361
Mongolian	30 (8)	8 (7)	10 (8)	12 (8)			
Tibetan	4 (1)	1 (1)	1 (1)	2 (1)			
Uyghurs Muslim	3 (1)	1 (1)	1 (1)	1 (1)			
aECOG performance status							
0	210 (54)	60 (55)	70 (51)	80 (54)	0.9918 (χ^2^-test)	6	0.81
1	161 (41)	42 (39)	56 (42)	63 (42)			
2	15 (4)	5 (5)	5 (4)	5 (3)			
3	4 (1)	1 (1)	1 (1)	2 (1)			

Continuous linear variables are depicted as mean ± Standard Deviation (SD).

Categorial variables are depicted as the frequencies with percentages in parenthesis.

a 0: Fully active, 1: Restricted in strenuous activity, and ≥ 2: Increasing disability. All results were significant if *p* < 0.05. ECOG, Eastern Cooperative Oncology Group; ANOVA, Analysis of variance; χ^2^-test, Chi-Square test for independence; df, Degree of freedom. Test value (F-value for ANOVA; χ^2^-value for χ^2^-test).

### Progression-free survival

**Fig. 2:**
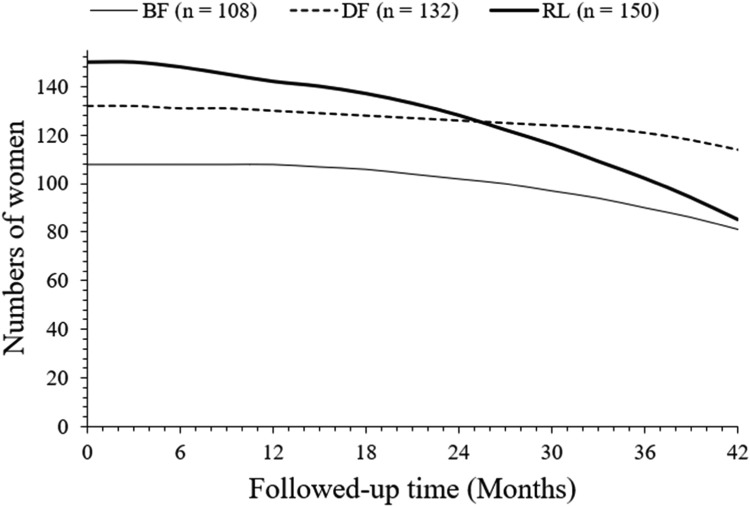
Progression-free survival of women. Progression-free survival: From the start of treatment(s) to the first documented progression of disease or death due to any reason.

After 42 months of follow-up, a total of 81 (75 %), 114 (88%), and 85 (57 %) women survived without progression in the BF, DF, and RL cohorts, respectively. After 42 months of follow-up, progression-free survived women were higher in the DF cohort than those in the BF (*p* = 0.0306, Fischer exact test, 95 % CI: 1.003 to 2.131 [using the approximation of Katz]) and RL (*p* < 0.0001, Fischer exact test, 95 % CI: 1.725 to 4.045 [using the approximation of Katz]). cohorts. After 42 months of follow-up, progression-free survived women were higher in the BF cohort than the RL cohort (*p* = 0.0025, Fischer exact test, 95 % CI: 1.168 to 2.367 [using the approximation of Katz]). The details of the progression-free survival of women are presented in Fig. 2. At 26 months, charts of progression-free survival of women in the DF and RL cohorts intercepted each other. However, the line art for the progression-free survival of women in the BF cohort in the progression-free survival of women chart is not intercepted to the line-art of the progression-free survival of women in the DF and RL cohorts.

### Overall survival

**Fig. 3:**
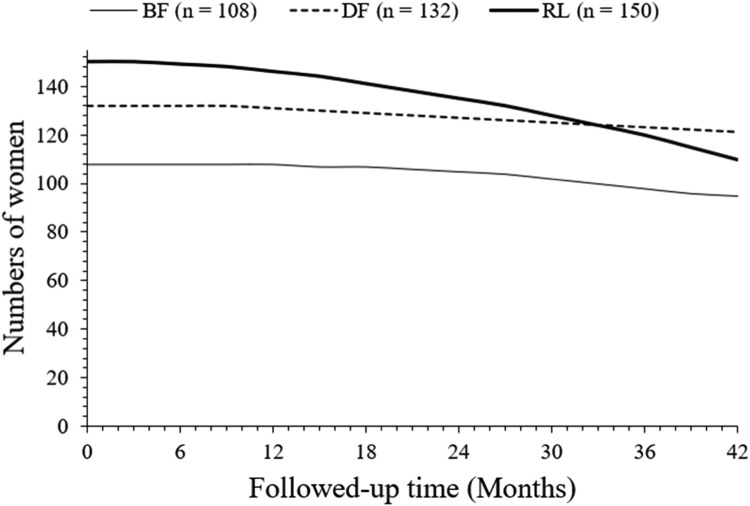
Survival of women. Survival: From the start of treatment(s) to death due to any reason.

After 42 months of follow-up, a total of 95 (88 %), 121 (92 %), and 110 (73 %) women survived in the BF, DF, and RL cohorts, respectively. Survival of women in the DF cohort was higher than that in the RL cohort (*p* < 0.0001, Fischer exact test, 95 % CI: 1.418 to 4.158 [using the approximation of Katz]). Survival of women in the DF cohort was higher than the BF cohort but was statistically not significant than that of women in the BF cohort (*p* = 0.3906, Fischer exact test, 95 % CI 0.7787 to 1.918 [using the approximation of Katz]). Survival of women in the BF cohort was higher than that in the RL cohort (*p* = 0.0047, Fisher's exact test, 95 % CI: 0.5824 to 0.8679 [using the approximation of Katz]). The details of the overall survival of women are presented in Fig. 3. At 33 months, overall survivals of women in the DF and RL cohorts intercepted each other. However, line art for the overall survival of women in the BF cohort in the overall survival of women chart is not intercepting to line art of the overall survival of women in the DF and RL cohorts.

### Clinical benefits

After treatment, 117 (89 %), 80 (74 %), and 84 (56 %) women from the BF, DF, and RL cohorts, respectively had clinical benefits. The clinical benefits for women in the DF cohort were greater than those in the BF and RL cohorts. The clinical benefits for women in the BF cohort were greater than those in the RL cohort. Women in the DF cohort had the highest clinical benefit, followed by women in the BF cohort, and women in the RL cohort had the least clinical benefit. Details of the clinical benefits to women after treatment(s) are presented in Table 2.

**Table 2:** Clinical benefits of women after treatment(s).

Parameters	Cohorts					Comparison between BF and RL
	DF	BF		RL		
Treatments	Dalpiciclib+fulvestrant	Buparlisib+fulvestrant		Ribociclib+letrozole		
Women	132	108	a*p*-value	150	a*p*-value	*p*-value
Women with clinical benefit	117 (89)	80 (74)	0.004 (95 % CI 1.114 to 2.603)	84 (56)	<0.0001 (95 % CI: 1.961 to 5.038)	0.0038 (95 % CI: 1.157 to 2.319)

Clinical benefits: The sum of women with complete response, partial response, and no signs of progressive response.

Variables are depicted as the frequencies with percentages in parenthesis.

a Concerning the DF value. Fischer exact test was used for statistical analyses. RECIST version 1.1 criteria was used for evaluation of clinical benefits. All results were significant if *p* < 0.05. 95 % CI, 95 % Confidence Interval (using the approximation of Katz.).

### Adverse effects

Patients in the DF, BF, and RL cohorts reported neutropenia, leukopenia, anemia, thrombocytopenia, and lymphopenia hematological adverse effects during treatment(s) and in the follow-up period. Neutropenia was more frequent in women of the DF and the BF cohorts than women in the RL cohort. Leukopenia was reported to be higher in women in the RL cohort than in those in the DF and BF cohorts. The details of the hematological adverse effects during treatment(s) and the follow-up period are reported in Table 3.

**Table 3:** Hematological adverse effects during treatment(s) and in the followed-up period.

Events	Cohorts							Comparison between BF and RL	
	DF	BF			RL				
Treatments	Dalpiciclib+fulvestrant	Buparlisib+fulvestrant			Ribociclib+letrozole				
Women	132	108	a*p*-value	95 % CI	150	a*p*-value	95 % CI	*p*-value	95 % CI
Neutropenia	115 (87)	99 (92)	0.301	0.6051 to 1.116	105 (70)^b^^,^^c^	0.0005	1.247 to 2.914	<0.0001	1.577 to 5.375
Leukopenia	114 (86)	98 (91)	0.3194	0.6178 to 1.133	142 (95)^b^	0.0221	0.4811 to 0.8601	0.2284	0.4731 to 1.142
Anemia	117 (89)	97 (90)	0.8367	0.6671 to 1.346	141 (94)	0.1349	0.5176 to 1.017	0.2431	0.4844 to 1.134
Thrombocytopenia	118 (89)	95 (88)	0.8379	0.7284 to 1.567	141 (94)	0.1926	0.5255 to 1.066	0.1133	0.4654 to 0.9971
Lymphopenia	119 (90)	94 (87)	0.539	0.7706 to 1.747	142 (95)	0.176	0.5134 to 1.057	0.041	0.4399 to 0.8906

CTCAE v5.0 was used for the evaluation of adverse events.

Women have one or more hematological adverse effect.

Variables are depicted as the frequencies with percentages in parenthesis.

a Concerning the DF value. Fischer exact test was used for statistical analyses. All results were significant if *p* < 0.05. 95 % CI, 95 % Confidence Interval (using the approximation of Katz.).

b Significant difference concerning the DF value.

c Significant difference concerning the BF value.

Patients in the DF, BF, and RL cohorts reported anorexia, headache, nausea, vomiting, hyperglycemia, skin rash, and fatigue as non-hematological adverse effects during treatment(s) and in the follow-up period. Vomiting, constipation, nausea, diarrhea, and anorexia were higher in women in the DF and BF cohorts than in women in the RL cohort. Increased levels of alanine aminotransferase and aspartate aminotransferase were reported to be higher in women in the RL cohort than in women in the DF and BF cohorts (*p* < 0.05, Fisher's exact test for both). Depression and anxiety were reported to be higher in women in the BF cohort than in those in the DF and RL cohorts (*p* < 0.05, Fisher's exact test for all). The details of non-hematological adverse effects during treatment(s) and in the follow-up period are reported in Table 4.

**Table 4:** Non-hematological adverse effects during treatment(s) and in the followed-up period.

Events	Cohorts								
	DF	BF			RL			Comparison between BF and RL	
Treatments	Dalpiciclib+fulvestrant	Buparlisib+fulvestrant			Ribociclib+letrozole				
Women	132	108	a*p*-value	95 % CI	150	a*p*-value	95 % CI	*p*-value	95 % CI
Headache	25 (19)	17 (16)	0.6092	0.8320 to 1.458	25 (17)	0.642	0.7950 to 1.478	0.8662	0.6448 to 1.432
Vomiting	27 (20)	22 (20)	0.9999	0.7549 to 1.331	21 (14)	0.1569	0.9407 to 1.671	0.1807	0.9151 to 1.788
Cough	14 (11)	11 (10)	0.9999	0.7061 to 1.474	16 (11)	0.9999	0.6650 to 1.494	0.9999	0.6006 to 1.567
Constipation	25 (19)	21 (19)	0.9999	0.7346 to 1.322	16 (11)	0.0622	1.035 to 1.822	0.0707	1.041 to 1.996
Insomnia	13 (10)	10 (9)	0.9999	0.7060 to 1.505	16 (11)	0.847	0.6234 to 1.457	0.8348	0.5473 to 1.515
Arthralgia	14 (11)	9 (8)	0.6612	0.7891 to 1.588	17 (11)	0.9999	0.6378 to 1.447	0.5312	0.4685 to 1.405
Back pain	15 (11)	12 (11)	0.9999	0.7065 to 1.448	20 (13)	0.7182	0.6037 to 1.356	0.7028	0.5504 to 1.416
Nausea	35 (27)	28 (26)	0.9999	0.7830 to 1.313	34 (23)	0.4892	0.8460 to 1.467	0.5579	0.8018 to 1.527
Hyperglycemia	22 (17)	27 (25)	0.147	0.5586 to 1.088	43 (29)^b^	0.023	0.4638 to 0.9613	0.5713	0.6383 to 1.256
Increased alanine aminotransferase	25 (19)	17 (16)	0.9999	0.7093 to 1.367	40 (27)^b^^,^^c^	0.0127	0.4293 to 0.9408	0.0475	0.4301 to 1.009
Increased aspartate aminotransferase	31 (23)	21 (19)	0.5293	0.8555 to 1.439	41 (27)	0.4955	0.6628 to 1.209	0.1836	0.5210 to 1.118
Diarrhea	35 (27)	35 (32)	0.3224	0.6702 to 1.146	35 (23)	0.5817	0.8289 to 1.441	0.1194	0.9586 to 1.730
Rash	25 (19)	25 (23)	0.43	0.6550 to 1.204	41 (27)	0.1209	0.5458 to 1.071	0.4728	0.6181 to 1.242
Fatigue	45 (34)	50 (46)	0.0636	0.6147 to 1.014	75 (50)^b^	0.008	0.5321 to 0.9163	0.614	0.6871 to 1.225
Depression	2 (1)	15 (14)^b^	0.0002	0.05462 to 0.7456	5 (3)^c^	0.454	0.1860 to 1.963	0.0035	1.424 to 2.588
Anxiety	5 (4)	20 (19)^b^	0.0002	0.1534 to 0.7476	10 (7)^c^	0.3039	0.3388 to 1.450	0.0052	1.278 to 2.335
Anorexia	62 (47)	85 (79)^b^	<0.0001	0.4487 to 0.6999	55 (37)^c^	0.0904	0.9762 to 1.598	<0.0001	2.108 to 4.602
Dysgeusia	5 (4)	20 (19)^b^	0.0002	0.1534 to 0.7476	14(9)^c^	0.0938	0.2541 to 1.169	0.0398	1.082 to 2.072

CTCAE v5.0 was used for the evaluation of adverse events.

Women have one or more hematological adverse effect.

Variables are depicted as the frequencies with percentages in parenthesis.

a Concerning the DF value. Fischer exact test was used for statistical analyses. All results were significant if *p* < 0.05. 95 % CI, 95 % Confidence Interval (using the approximation of Katz.).

b Significant difference concerning the DF value.

c Significant difference concerning the BF value.

## Discussion

The study reported that postmenopausal women with hormone receptor-positive and HER2-negative breast cancer who received dalpiciclib plus fulvestrant had higher progression-free survival, overall survival, and clinical benefits than postmenopausal women with hormone receptor-positive and HER2-negative breast cancer who received buparlisib plus fulvestrant or ribociclib plus letrozole. Dalpiciclib provides extended benefits of cure from diseases (breast cancer) compared to buparlisib or ribociclib plus letrozole,^8^ because dalpiciclib has dose-dependent plasma exposure in Chinese women with hormone receptor-positive and HER2-negative breast cancer.^17^ The results of this study suggest that dalpiciclib plus fulvestrant is effective in postmenopausal Chinese women with hormone receptor-positive and HER2-negative breast cancer.

Women who received dalpiciclib or buparlisib reported neutropenia during treatment and follow-up periods. The results of the hematological adverse effects of the current study are consistent with those of a phase 3 trial^8^ and a phase 1 trial.^17^ CDK4/6 inhibitors have adverse effects on neutropenia in Chinese women.^18^ Dalpiciclib and buparlisib cause neutropenia.

Women who received dalpiciclib or buparlisib plus fulvestrant reported non-hematological adverse effects related to the gastrointestinal tract during the treatment and follow-up periods. Fulvestrant is responsible for adverse effects in the gastrointestinal tract.^19^ It is necessary to manage adverse effects related to the gastrointestinal tract during treatment with fulvestrant.

Increased aspartate aminotransferase levels were reported to be higher in women in the RL cohort during the treatment and follow-up periods. The results of the hepatological adverse effects of the current study are consistent with those of a phase 3 trial^5^ and a MONALEESA-2 trial.^6^ Liver function monitoring is recommended for ribociclib plus letrozole treatment in postmenopausal women with hormone receptor-positive and HER2-negative breast cancers.

In the women in the BF cohort, skin rashes, diarrhea, and increased levels of alanine aminotransferase and aspartate aminotransferase were reported. The adverse effects of buparlisib in the current study were consistent with those in a phase I trial.^13^ Daily buparlisib (100 mg) was responsible for the adverse effects.

Women in the BF cohort had higher levels of depression and anxiety during treatment and follow-up periods. The results of the psychiatric adverse effects in the current study are consistent with those of a phase 3 trial.^10^ The highly penetrating properties of the blood-brain barrier of buparlisib are responsible for anxiety and depression.^11^ During treatment with buparlisib, women should be under the supervision of a consultant.

The current study has several limitations, for example, it is a retrospective study and lacks randomized trials. The study was preliminary, and the discriminating criteria of the treatment were not introduced. More demographic and clinical parameters should be considered and be well-balanced. The statistical analysis for Cox regression of the primary outcomes in the manuscript, treatment options, ECOG status, and safety and efficacy of treatment was not performed.

## Conclusions

Dalpiciclib plus fulvestrant is more effective and comparatively safe (than fulvestrant plus buparlisib treatment and ribociclib plus letrozole treatment) in postmenopausal women with hormone receptor-positive and HER2-negative breast cancers. Dalpiciclib and buparlisib caused neutropenia during the treatment and follow-up periods. It is necessary to manage the adverse effects related to the gastrointestinal tract during treatment with fulvestrant and follow-up periods. Liver function monitoring is recommended for ribociclib plus letrozole treatment during treatment and follow-up periods in postmenopausal women with hormone receptor-positive and HER2-negative breast cancer. Daily buparlisib (100 mg) was responsible for the adverse effects.

## Availability of data and materials

The datasets used and analyzed during the current study are available from the corresponding author upon reasonable request.

## CRediT authorship contribution statement

**Qi Liu:** Project administration, Conceptualization, Formal analysis, Supervision, Resources, Methodology, Validation, Writing – review & editing, Visualization, Data curation. **Lingli Hou:** Investigation, Resources, Conceptualization, Visualization, Formal analysis, Methodology, Writing – review & editing, Validation, Data curation. **Ying Zhao:** Resources, Formal analysis, Conceptualization, Methodology, Data curation, Writing – review & editing, Validation, Visualization. **Hongwei Yang:** Resources, Supervision, Formal analysis, Data curation, Writing – review & editing, Validation, Visualization. **Zhengying Mo:** Formal analysis, Funding acquisition, Resources, Data curation, Software, Writing – review & editing, Visualization, Validation. **Fei Yu:** Resources, Conceptualization, Formal analysis, Methodology, Writing – review & editing, Writing – original draft, Validation, Visualization.

## Conflicts of Interest

The authors declare no conflicts of interest.
